# Full-Reference Image Quality Assessment Based on an Optimal Linear Combination of Quality Measures Selected by Simulated Annealing

**DOI:** 10.3390/jimaging8080224

**Published:** 2022-08-21

**Authors:** Domonkos Varga

**Affiliations:** Ronin Institute, Montclair, NJ 07043, USA; domonkos.varga@ronininstitute.org

**Keywords:** full-reference image quality assessment, feature selection, simulated annealing

## Abstract

Digital images can be distorted or contaminated by noise in various steps of image acquisition, transmission, and storage. Thus, the research of such algorithms, which can evaluate the perceptual quality of digital images consistent with human quality judgement, is a hot topic in the literature. In this study, an image quality assessment (IQA) method is introduced that predicts the perceptual quality of a digital image by optimally combining several IQA metrics. To be more specific, an optimization problem is defined first using the weighted sum of a few IQA metrics. Subsequently, the optimal values of the weights are determined by minimizing the root mean square error between the predicted and ground-truth scores using the simulated annealing algorithm. The resulted optimization-based IQA metrics were assessed and compared to other state-of-the-art methods on four large, widely applied benchmark IQA databases. The numerical results empirically corroborate that the proposed approach is able to surpass other competing IQA methods.

## 1. Introduction

Nowadays, people increasingly communicate through media in form of audio, video, and digital images. Therefore, image quality assessment (IQA) has found many applications and become a hot research topic in the research community [1]. Namely, IQA methods evaluate the perceptual quality of digital images and support, among others, image enhancement [2], restoration [3], steganography [4], or denoising algorithms [5]. Further, IQA is also necessary in the benchmarking of many image processing or computer-vision algorithms [6,7,8]. In the literature, IQA is classified into two groups, i.e., subjective and objective IQA. Specifically, subjective IQA deals with the collection of users’ quality ratings for a set of digital images either in a laboratory [1] or in an online crowd-sourcing experiment [9]. Moreover, images’ perceptual quality is expressed as a mean opinion score (MOS), which is the arithmetic mean of individual quality scores. As a result, subjective IQA provides quality labelled images with objective IQA as training or test data [10]. Namely, objective IQA deals with algorithms and mathematical models that are able to predict the quality of a given image. Conventionally, objective IQA is divided into three classes [11]—full-reference (FR) [12], reduced-reference (RR) [13], and no-reference (NR) [14]—with respect to the availability of the reference (distortion-free) images. As the names indicate, FR-IQA methods have full access to the reference images. In contrast, NR-IQA algorithms evaluate image quality without any information about the reference images [15], and RR-IQA algorithms have partial information about them.

### 1.1. Contribution

The development of objective FR-IQA algorithms can also involve fusion-based strategies that already take existing FR-IQA metrics and try to create a “super evaluator”. Recently, many complex fusion-based approaches have been published in the literature [16,17,18,19]. The main contribution to this paper is also a fusion-based approach. Namely, we demonstrate a solution based on a linear combination of several already existing FR-IQA metrics optimized with a simulated annealing (SA) algorithm using a root mean square error (RMSE) objective, which is able to produce well-performing fusion-based FR-IQA metrics. To be more specific, a linear combination of 16 FR-IQA metrics is used in an optimization problem to select FR-IQA metrics and find their weights via an SA algorithm that minimizes the RMSE of the prediction. Unlike the approach of Oszust [20], we apply simulated annealing instead of a genetic algorithm for performing the fusion of FR-IQA metrics. Namely, simulated annealing usually achieves better results in the case of continuous function approximation than basic genetic algorithms because they choose one or two genes at a given location [21]. The proposed fusion-based metrics was evaluated on large, popular, and widely accepted IQA benchmark databases, such as LIVE [22], TID2013 [23], TID2008 [24], and CSIQ [25].

### 1.2. Organization

The rest of this paper is organized as follows. In Section 2, an overview about the current state of FR-IQA is given. Next, the proposed fusion-based metric is introduced in Section 3. Our experimental results, together with the description of the applied benchmark IQA databases, evaluation environment, and performance indices, are given in Section 4. Finally, a conclusion is drawn in Section 5.

## 2. Literature Review

In this paper, we follow the classification of FR-IQA algorithms presented in [26]. To be specific, Ding et al. [26] categorized existing FR-IQA algorithms into five distinct classes, i.e., error visibility, structural similarity, information theoretic, learning-based, and fusion-based methods.

Error visibility methods measure a distance between the pixels of the distorted and the reference images to quantify perceptual quality degradation. The representative method of this class of FR-IQA is the mean squared error (MSE) method, which measures the average of the squares of the errors. In other words, it is the average squared difference between the reference and the distorted images in the context of FR-IQA [27]. Another well-known example is the peak signal-to-noise ratio (PSNR), which is commonly applied to assess the quality of the reconstruction of lossy compression codecs [28]. Although both MSE and PSNR have low computational costs and their physical meaning is clear and well understood, they often mismatch with subjective perceptions of visual quality.

Structural similarity methods measure the similarity between the corresponding regions of the distorted and reference images using sliding-windows in the images and correlation measures. The representative and first published method of this class is the structural similarity index (SSIM) [29], which has become extremely popular in the field with many extensions and applications [30]. The theorem of SSIM has become extremely popular in the research community and inspired many variants. For example, the wavelet domain structural similarity [31] carries out SSIM in the wavelet domain to quantify perceptual quality. This work was extended by Sampat et al. [32] into the complex wavelet domain. In [33], information content was utilized as weights in the pooling process of local image quality scores. In contrast, Wang et al. [34] extended SSIM to multi-scale processing to improve perceptual quality estimation. Li and Bovik [35] elaborated an FR-IQA metric by taking the average of SSIMs computed over three different regions of an image, such as edges, textures, and smooth regions. Kolaman and Yadid-Pecht [36] found an extension of SSIM to color images by representing red, green, and blue color channels with quaternions. Later, SSIM was also extended to hyperspectral images [37].

Information theoretic methods approach the FR-IQA task from the point of view of information communication. For example, Sheikh et al. [38,39] compared the information content of the reference and distorted images. Namely, perceptual quality was quantified by how much information is similar between the reference and distorted images. In contrast, Larson and Chandler [25] classified image distortions as near-threshold and supra-threshold. The authors elaborated two quality indexes for both distortion types. Finally, the overall perceptual quality was determined based on the quality scores of near-threshold and supra-threshold distortions.

As the terminology suggests, learning-based methods rely on a specific machine learning algorithm to create a quality model from training images. Next, the obtained quality model is tested on previously unseen images. For instance, Liang et al. [40] implemented a special convolutional neural network containing two paths, one for the reference image and the other for the distorted image. Further, this network was trained on 224×224-sized image patches sampled simultaneously from the reference and distorted images. As a consequence, the perceptual quality of a distorted image was estimated by the average score of the considered patches. Kim and Lee [41] devised a similar network, but it predicts a visual sensitivity map that is multiplied by an error map calculated directly from the reference and the distorted images to estimate perceptual image quality. Ahn et al. [42] further improved the idea of Kim and Lee [41] by implementing an end-to-end trained convolutional neural network with three inputs, i.e., reference image, distorted image, and spatial error map. Similar to [41], a distortion-sensitivity map was predicted from the inputs and was later multiplied by the spatial error map to give an estimation for the perceptual image quality. In contrast to the previously mentioned methods, Ding et al. [43] extracted a set of feature maps from the reference and the distorted images using the Sobel operator, log Gabor filter, and local pattern analysis. Subsequently, the extracted feature maps were compared, and from the resulting similarity scores a feature vector was compiled that was mapped onto perceptual quality scores with a trained support vector regressor. Tang et al. [44] took a similar approach, but the authors employed a different set of features (phase congruency maps [45], gradient magnitude maps, and log Gabor maps). Further, the similarity scores of the feature maps were mapped onto perceptual quality with a trained random forest regressor.

Fusion-based FR-IQA methods utilize existing FR-IQA metrics to create a new FR-IQA algorithm. First, Okarma [46] suggested the idea of combined methods. Namely, the author proposed a combined metric using the product and power of MS-SSIM [34], VIF [38], and R-SVD [47]. This approach was developed further in [19], where the optimal exponents in the product were determined by using MATLAB’s fminsearch command. In [48], Oszust took a similar approach, but the author applied the scores of traditional FR-IQA metrics as predictor variables in a lasso regression. Instead of lasso regression, Yuan et al. [49] used kernel ridge regression in a similar layout. The work of Lukin et al. [50] exhibits the properties of both learning-based and fusion-based methods. Specifically, the authors created a training and a test set from the images of an IQA benchmark database. Next, the scores of several traditional FR-IQA metrics were used as image features, and a neural network was trained to estimate perceptual image quality. Amirshahi et al. [51] elaborated a special fusion-based FR-IQA metric relying on a pretrained convolutional neural network. Namely, the authors ran a reference-distorted image pair through an AlexNet [52] network and compared the activation maps with the help of a traditional FR-IQA metric. Next, the resulted scores were aggregated to obtain a single score for the perceptual image quality. Bakurov et al. [53] revisited the classical SSIM [29] and MS-SSIM [34] metrics by applying evolutionary and swarm intelligence optimization methods to find optimal hyperparameters for SSIM and MS-SSIM instead of the original settings. Fusion-based metrics were also proposed for remote sensing images [54], stitched panoramic images [55], and 3D image quality assessment [18].

For more detailed studies about FR-IQA, we refer readers to the book of Xu et al.’s [56] and to the study of Pedersen and Hardeberg [57]. Further, Zhang et al. [58] provide an evaluation of several state-of-the-art FR-IQA algorithms on various IQA benchmark databases. Zhai and Min provided an comprehensive overview of classical algorithms in [59]. For the quality assessment of screen content images [60], Min et al. gave an overview in [61].

## 3. Proposed Method

As already mentioned, an FR-IQA metric should deliver perceptual quality scores consistent with the human judgement using both the distorted and reference images. Let us express the aggregated decision of *n* different FR-IQA metrics by a weighted sum as:

(1) $$Q=\sum_{i=1}^{n}\alpha_{i}q_{i},$$

where $q_{i}\left(i=1,2,...,n\right)$ stands for the quality scores provided by the FR-IQA metrics. Further, $\alpha =(\alpha_{1},\alpha_{2},\ldots ,\alpha_{n})$ is a real vector of weights whose values are found via an optimization procedure to ensure an effective fusion of FR-IQA metrics. Namely, an optimization fusion was carried out in our study using n=16 open-source FR-IQA metrics, such as FSIM [62], FSIMc [62], GSM [63], IFC [38], IFS [64], IW-SSIM [33], MAD [25], MS-SSIM [34], NQM [65], PSNR, RFSIM [66], SFF [67], SR-SIM [12], SSIM [29], VIF [39], and VSI [68].

**Figure 1:**
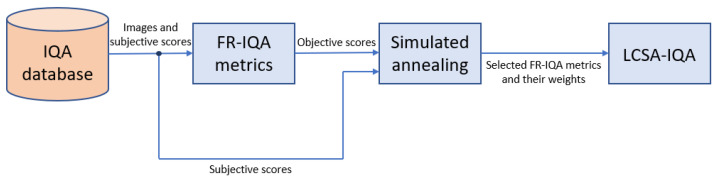
In the offline optimization stage, the proposed fusion-based metric is obtained by using 20% of the reference with its corresponding distorted counterparts. Next, a simulated annealing (SA) optimization process selects FR-IQA metrics and provides them with weights. The resulting metric is codenamed as LCSA-IQA to refer to the fact that is the linear combination of selected FR-IQA metrics where the weights were assigned using simulated annealing.

**Figure 2:**
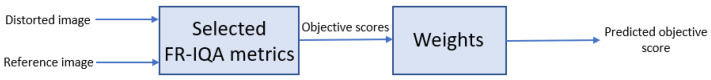
The optimal linear combination of the selected FR-IQA metrics is applied to estimate perceptual image quality.

In the literature, Pearson’s linear correlation coefficient (PLCC), Spearman’s rank-order correlation coefficient (SROCC), Kendall’s rank order correlation coefficient (KROCC), and root mean square error (RMSE) are often considered to characterize the consistency between the ground-truth quality scores of an IQA benchmark database and the quality scores predicted by an FR-IQA metric [22]. From these performance indices, RMSE was applied as an objective function in the proposed optimization based metric. Figure 1 and Figure 2 depict flowcharts where the compilation of the proposed fusion-based metrics and its application for FR-IQA are demonstrated.

Formally, the optimization problem can be written as

(2) $$\begin{array}{c} \min_{\alpha }RMSE\left(F\left(Q_{p},\beta \right),S\right),\\ \mathrm{subject}\;\mathrm{to}\;\alpha_{i}\in R,n\in N,\beta \geq 0, \end{array}$$

where $Q_{p}$ is vector containing the quality scores of a set of images obtained by Equation (1) and S contains the corresponding ground-truth scores. Further, prior to the calculation of RMSE, a non-linear regression is also applied [22] since a non-linear relationship exists between the ground-truth and predicted scores. Formally, it can be written

(3) $$Q=\beta_{1}\left(\frac{1}{2}-\frac{1}{1+e^{\beta_{2}\left(Q_{p}-\beta_{3}\right)}}\right)+\beta_{4}Q_{p}+\beta_{5},$$

where $\beta_{1},...,\beta_{5}$ stand for the parameters of the regression model. In addition, *Q* and $Q_{p}$ are the fitted and predicted scores, respectively. Since we use four large, widely accepted IQA benchmark databases, i.e., LIVE [22], TID2013 [23], TID2008 [24], and CSIQ [25], in this paper, four optimization-based fusion FR-IQA metrics are proposed, respectively. To this end, approximately 20% of the reference images were randomly selected from a given benchmark IQA database. More precisely, Q and S were compiled based on those distorted images whose reference counterparts were randomly selected. Although 20% is a common choice for parameter setting in the literature [69,70], there are also researchers who applied 30% [62] or 80% [71] for parameter tuning. However, we evaluate all the fusion based metrics on all the databases to demonstrate results independent from the database.

Next, the optimization problem was solved described by Equation (2) to determine the $\alpha_{i}$ weights for Equation (1). Since the number of possible solutions increases exponentially with number of the considered FR-IQA metrics, simulated annealing (SA) [72,73] was used to solve the above-described optimization task. Namely, SA is a probabilistic optimization technique for estimating the global optimum of a given function. The stochastic nature of this algorithm enables the usage of nonlinear objective functions where many other methods do not operate well. SA was inspired by the physical model of heating a material and then slowly decreasing the temperature to eliminate imperfections from the material. Hence, minimizing the system’s energy is the main goal. More precisely, the SA randomly generates a new point at each iteration. Based on a probability distribution with a scale proportional to the temperature, the new point’s distance from the present point or the size of the search is determined. All new points that reduce the objective are accepted by the algorithm, but points that increase the objective can also be accepted with a pre-defined probability. Due to this property of the method, SA is prevented from being stuck in local minima in early iterations. In our implementation, the SA was performed using MATLAB R2020a with a Global Optimization Toolbox using $\alpha_{i}=0$ for i=1,2,…,n as initial point and defining no lower or upper bounds for the method. After 100 runs of SA, the best solution— $\alpha_{d}^{best}$—was selected, where *d* denotes the database from which 20% of the reference images was chosen randomly.

In the end of the SA optimization processes using LIVE [22], TID2013 [23], TID2008 [24], and CSIQ [25] databases, the following FR-IQA metrics can be obtained, which are codenamed LCSA, referring to the fact that they are linear combinations of FR-IQA measures selected by simulated annealing:

(4) $$\begin{array}{c} LCSA1=\alpha_{\mathrm{LIVE}}^{\mathrm{best}}=-561.0123\cdot VSI+281.826\cdot FSIMc-116.1501\cdot IFC-846.6376\cdot MAD\\ +349.6191\cdot MSSSIM-262.6766\cdot NQM+41.6348\cdot PSNR-308.9426\cdot SSIM\\ +722.4479\cdot VIF, \end{array}$$

(5) $$\begin{array}{c} LCSA2=\alpha_{\mathrm{TID}2013}^{\mathrm{best}}=1774.8368\cdot VSI+467.5433\cdot FSIMc-332.1863\cdot GSM-63.4379\cdot IFC\\ +84.7954\cdot IWSSIM-346.5585\cdot MAD-126.5188\cdot NQM+381.0923\cdot PSNR\\ -626.9841\cdot SSIM+380.3341\cdot VIF+524.6484\cdot IFS+342.7968\cdot SFF, \end{array}$$

(6) $$\begin{array}{c} LCSA3=\alpha_{\mathrm{TID}2008}^{\mathrm{best}}=1253.2402\cdot VSI+217.0877\cdot IWSSIM-168.1779\cdot MAD\\ -75.6832\cdot NQM+276.9035\cdot PSNR-28.5915\cdot RFSIM-454.7619\cdot SSIM+203.0893\cdot VIF\\ +500.4323\cdot IFS-153.3686\cdot SFF, \end{array}$$

(7) $$\begin{array}{c} LCSA4=\alpha_{\mathrm{CSIQ}}^{\mathrm{best}}=266.3256\cdot FSIM-119.8937\cdot FSIMc-15.6937\cdot IWSSIM\\ -529.1806\cdot MAD-656.4991\cdot MSSSIM-73.009\cdot NQM+381.0923\cdot PSNR\\ -626.9841\cdot SSIM+380.3341\cdot VIF+524.6484\cdot IFS+342.7968\cdot SFF. \end{array}$$

The corresponding β vectors are as follows:

(8) $$\beta_{\mathrm{LIVE}}=\left(106.1735,36.8421,30.0447,15.7705,139.3613\right),$$

(9) $$\beta_{\mathrm{TID}2013}=\left(56.413,193.7249,14.9834,147.7736,89.8778\right),$$

(10) $$\beta_{\mathrm{TID}2008}=\left(13.4153,115.9834,45.4464,22.0253,269.7624\right),$$

(11) $$\beta_{\mathrm{CSIQ}}=\left(13.5361,105.4132,70.1095,150.7645,11.5291\right).$$

## 4. Results

In this section, our experimental results are presented. First, the applied IQA benchmark databases and evaluation protocol are described in Section 4.1. Next, Section 4.2 presents a comparison to other competing state-of-the-art methods on four large IQA benchmark databases, i.e., LIVE [22], TID2013 [23], TID2008 [24], and CSIQ [25].

### 4.1. Applied IQA Benchmark Databases and Evaluation Protocol

**Figure 3:**
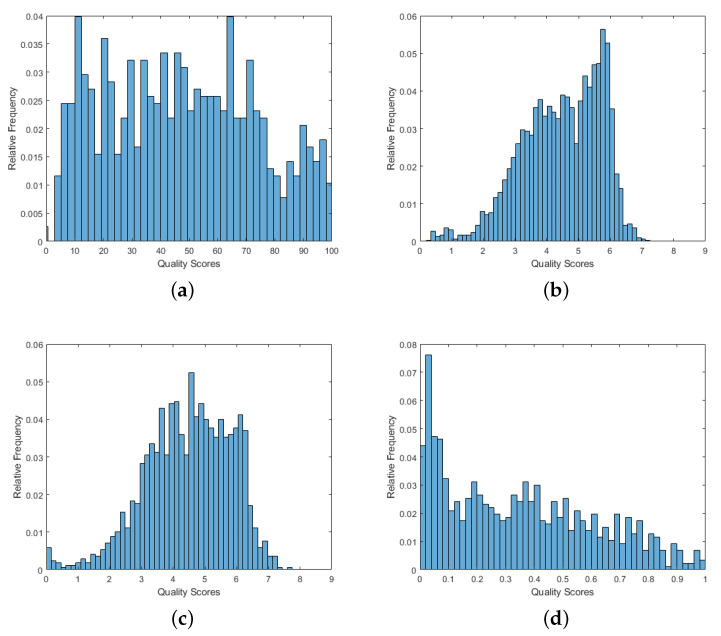
Empirical MOS distributions in the used benchmark IQA databases: (**a**) LIVE, (**b**) TID2013, (**c**) TID2008, and (**d**) CSIQ.

The main properties of the applied IQA benchmark databases are outlined in Table 1. These databases consist of a set of reference images, whose visual quality are considered perfect and flawless. Further, distorted images are generated artificially from the reference images using different distortion types (i.e., JPEG compression noise, JPEG2000 compression noise, salt and pepper, motion blur, Gaussian, Poisson, etc.) at different distortion levels. Figure 3 depicts the empirical MOS distributions of the applied benchmark databases.

In the literature, PLCC, SROCC, and KROCC is widely used and accepted to characterize the performance of FR-IQA methods. They are measured between the ground-truth scores of an IQA benchmark database and the predicted scores. Moreover, prior to the calculation of PLCC a non-linear regression is also applied [22] since a non-linear relationship exists between the ground-truth and predicted scores. This non-linear relationship was also defined by Equation (3). Further, *Q* and $Q_{p}$ are the fitted and predicted scores, respectively. PLCC between vectors **x** and **y** with length *m* is defined as

(12) $$PLCC\left(x,y\right)=\frac{x^{T}y}{\sqrt{{\bar{x}}^{T}\bar{y}{\bar{y}}^{T}\bar{x}}},$$

where $\bar{x}$ and $\bar{y}$ are the mean subtracted version of vectors x and y, respectively. On the other hand, SROCC can be defined as

(13) $$SROCC\left(x,y\right)=1-\frac{6\sum_{i=1}^{m}{\left(x_{i}-y_{i}\right)}^{2}}{m(m^{2}-1)},$$

where $x_{i}$ and $y_{i}$ are the *i*th entries of vectors **x** and **y**, respectively. In contrast, KROCC uses the number of concordant pairs ($m_{c}$) and the number of discordant pairs ($m_{d}$) between vectors **x** and **y** and is defined as

(14) $$KROCC\left(x,y\right)=\frac{m_{c}-m_{d}}{\frac{1}{2}m\left(m-1\right)}.$$

As already mentioned, the proposed fusion-based metrics were implemented using MATLAB R2020a and its Global Optimization Toolbox. The computer configuration applied in our experiments is summarized in Table 2.

### 4.2. Comparison to the State-of-the-Art

In this subsection, the proposed fusion-based metrics are compared to several state-of-the-art FR-IQA whose original source codes were made publicly available by the authors. Moreover, we reimplemented the fusion-based SSIM-CNN [51] method in MATLAB R2020a (available at: https://github.com/Skythianos/SSIM-CNN (accessed on 12 May 2022)). The PLCC, SROCC, and KROCC performance comparisons of the proposed fusion-based FR-IQA metrics with the state-of-the-art are summarized in Table 3 and Table 4. Specifically, Table 3 demonstrates the results on LIVE [22] and TID2013 [23], while Table 4 contains the obtained results for TID2008 [24] and CSIQ [25] databases. The obtained results clearly show that the proposed LCSA metrics are able to outperform the state-of-the-art. Specifically, those LCSA metrics that were parameter-tuned on database *d* always deliver the highest correlation values, while another LCSA not parameter-tuned on database *d* usually provides the second-best results.

Table 5 illustrates the direct and weighted average of correlation values measured on LIVE [22], TID2013 [23], TID2008 [24], and CSIQ [25]. From the results of direct averages, it can be clearly seen that the proposed LCSA2 and LCSA4 provide the best results in two out of three performance indices, while LCSA3 is able to produce second best KROCC value. The results of weighted averages are biased towards those FR-IQA measures that perform well on TID2013 [23] since it is the largest database from the applied benchmarks. Similarly, LCSA2 is the best-performing method in this respect because it provides the best results for SROCC and KROCC. Further, LCSA4 delivers the second best PLCC and KROCC values, while LCSA3’s performance is equivalent in terms of SROCC and KROCC to those of LCSA4.

In the following, we examine the performance of the proposed and the other state-of-the-art methods on the individual distortion types of the applied IQA benchmark databases. The distortion types and their abbreviations used by the databases are summarized in Table 6. Further, Table 7, Table 8, Table 9 and Table 10 contain detailed results on the different distortion types of LIVE [22], TID2013 [23], TID2008 [24], and CSIQ [25], respectively. To be more specific, the SROCC values are given for each individual distortion types.

## 5. Conclusions

In this study, we presented a novel fusion-based FR-IQA metric using simulated annealing. Specifically, an optimization problem was solved based on the weighted sum of several FR-IQA metrics by minimizing the root mean squared error between the predicted and ground-truth perceptual quality scores. The evaluation of the proposed fusion-based metrics on four large publicly available and widely accepted IQA benchmark databases empirically corroborated that the proposed metrics are able to produce competitive results compared to the state-of-the-art in terms of various performance indices, such as PLCC, SROCC, and KROCC. Future research could involve other optimization techniques and their combination for improved perceptual quality prediction. Another direction is the generalization of the proposed method for other types of media.

## Figures and Tables

**Table 1:** Summary of benchmark databases used in this study.

	LIVE [22]	TID2013 [23]	TID2008 [24]	CSIQ [25]
No. of reference images	29	25	25	30
No. of distorted images	779	3000	1700	866
No. of distortions	5	24	17	6
No. of levels	5	5	4	4-5
No. of observers	161	917	838	35
Resolution	∼768×512	512×384	512×384	500×500

**Table 2:** Computer configuration applied in our experiments.

Computer model	STRIX Z270H Gaming
Operating system	Windows 10
Memory	15 GB
CPU	Intel(R) Core(TM) i7-7700K CPU 4.20 GHz (8 cores)
GPU	Nvidia GeForce GTX 1080

**Table 3:** PLCC, SROCC, and KROCC performance comparison of the proposed fusion-based FR-IQA metrics on LIVE and TID2013 databases with the state-of-the-art. The best results are typed in bold, and the second best results are underlined.

	LIVE [22]			TID2013 [23]		
**FR-IQA Metric**	**PLCC**	**SROCC**	**KROCC**	**PLCC**	**SROCC**	**KROCC**
2stepQA [74]	0.937	0.932	0.828	0.736	0.733	0.550
CSV [75]	0.967	0.959	0.834	0.852	0.848	0.657
DISTS [76]	0.954	0.954	0.811	0.759	0.711	0.524
ESSIM [77]	0.963	0.962	0.840	0.740	0.797	0.627
FSIM [62]	0.960	0.963	0.833	0.859	0.802	0.629
FSIMc [62]	0.961	0.965	0.836	0.877	0.851	0.667
GSM [63]	0.944	0.955	0.831	0.789	0.787	0.593
IFC [38]	0.927	0.926	0.758	0.554	0.539	0.394
IFS [64]	0.959	0.960	0.825	0.879	0.870	0.679
IW-SSIM [33]	0.952	0.956	0.817	0.832	0.778	0.598
MAD [25]	0.967	0.967	0.842	0.827	0.778	0.600
MS-SSIM [34]	0.941	0.951	0.804	0.794	0.785	0.604
NQM [65]	0.912	0.909	0.741	0.690	0.643	0.474
PSNR	0.872	0.876	0.687	0.616	0.646	0.467
ReSIFT [78]	0.961	0.962	0.838	0.630	0.623	0.471
RFSIM [66]	0.935	0.940	0.782	0.833	0.774	0.595
RVSIM [79]	0.641	0.630	0.495	0.763	0.683	0.520
SFF [67]	0.963	0.965	0.836	0.871	0.851	0.658
SR-SIM [12]	0.955	0.962	0.829	0.859	0.800	0.631
SSIM [29]	0.941	0.951	0.804	0.618	0.616	0.437
SSIM-CNN [51]	0.965	0.963	0.838	0.759	0.752	0.566
SUMMER [80]	0.967	0.959	0.833	0.623	0.622	0.472
VIF [39]	0.941	0.964	0.828	0.774	0.677	0.515
VSI [68]	0.948	0.952	0.805	0.900	0.894	0.677
*LCSA1*	**0.974**	**0.974**	**0.857**	0.820	0.788	0.607
*LCSA2*	0.846	0.962	0.828	**0.916**	**0.903**	**0.731**
*LCSA3*	0.947	0.969	0.843	0.770	0.821	0.647
*LCSA4*	0.967	0.970	0.844	0.859	0.823	0.649

**Table 4:** PLCC, SROCC, and KROCC performance comparison of the proposed fusion-based FR-IQA metrics on TID2008 and CSIQ databases with the state-of-the-art. The best results are typed in bold, and the second best results are underlined.

	TID2008 [24]			CSIQ [25]		
**FR-IQA Metric**	**PLCC**	**SROCC**	**KROCC**	**PLCC**	**SROCC**	**KROCC**
2stepQA [74]	0.757	0.769	0.574	0.841	0.849	0.655
CSV [75]	0.852	0.848	0.657	0.933	0.933	0.766
DISTS [76]	0.705	0.668	0.488	0.930	0.930	0.764
ESSIM [77]	0.658	0.876	0.696	0.814	0.933	0.768
FSIM [62]	0.874	0.881	0.695	0.912	0.924	0.757
FSIMc [62]	0.876	0.884	0.699	0.919	0.931	0.769
GSM [63]	0.782	0.781	0.578	0.896	0.911	0.737
IFC [38]	0.575	0.568	0.424	0.837	0.767	0.590
IFS [64]	0.879	0.869	0.678	0.958	0.958	0.817
IW-SSIM [33]	0.842	0.856	0.664	0.804	0.921	0.753
MAD [25]	0.831	0.829	0.639	0.950	0.947	0.797
MS-SSIM [34]	0.838	0.846	0.648	0.899	0.913	0.739
NQM [65]	0.608	0.624	0.461	0.743	0.740	0.564
PSNR	0.447	0.489	0.346	0.853	0.809	0.599
ReSIFT [78]	0.627	0.632	0.484	0.884	0.868	0.695
RFSIM [66]	0.865	0.868	0.678	0.912	0.930	0.765
RVSIM [79]	0.789	0.743	0.566	0.923	0.903	0.728
SFF [67]	0.871	0.851	0.658	0.964	0.960	0.826
SR-SIM [12]	0.859	0.799	0.631	0.925	0.932	0.773
SSIM [29]	0.669	0.675	0.485	0.812	0.812	0.606
SSIM-CNN [51]	0.770	0.737	0.551	0.952	0.946	0.794
SUMMER [80]	0.817	0.823	0.623	0.826	0.830	0.658
VIF [39]	0.808	0.749	0.586	0.928	0.920	0.754
VSI [68]	0.898	0.896	0.709	0.928	0.942	0.785
*LCSA1*	0.886	0.874	0.685	0.966	0.956	0.819
*LCSA2*	0.896	0.906	0.727	0.897	0.949	0.800
*LCSA3*	**0.923**	**0.921**	**0.755**	0.964	0.961	0.827
*LCSA4*	0.906	0.909	0.737	**0.977**	**0.973**	**0.857**

**Table 5:** PLCC, SROCC, and KROCC performance comparison of the proposed fusion-based FR-IQA metrics with the state-of-the-art. The best results are typed in bold, the second best results are underlined.

	Direct Average			Weighted Average		
**FR-IQA Metric**	**PLCC**	**SROCC**	**KROCC**	**PLCC**	**SROCC**	**KROCC**
2stepQA [74]	0.818	0.821	0.652	0.781	0.783	0.605
CSV [75]	0.901	0.897	0.729	0.877	0.873	0.694
DISTS [76]	0.837	0.816	0.647	0.792	0.759	0.582
ESSIM [77]	0.794	0.892	0.733	0.756	0.857	0.691
FSIM [62]	0.901	0.893	0.729	0.883	0.860	0.689
FSIMc [62]	0.908	0.908	0.743	0.893	0.885	0.710
GSM [63]	0.853	0.859	0.685	0.821	0.823	0.638
IFC [38]	0.723	0.700	0.542	0.644	0.625	0.473
IFS [64]	0.919	0.914	0.750	0.900	0.893	0.715
IW-SSIM [33]	0.857	0.878	0.708	0.846	0.840	0.664
MAD [25]	0.894	0.880	0.720	0.862	0.838	0.667
MS-SSIM [34]	0.868	0.874	0.699	0.838	0.839	0.659
NQM [65]	0.738	0.729	0.560	0.703	0.684	0.516
PSNR	0.697	0.705	0.525	0.634	0.654	0.480
ReSIFT [78]	0.776	0.771	0.622	0.705	0.700	0.550
RFSIM [66]	0.886	0.878	0.705	0.865	0.841	0.663
RVSIM [79]	0.779	0.740	0.577	0.777	0.723	0.558
SFF [67]	0.917	0.908	0.745	0.895	0.880	0.703
SR-SIM [12]	0.900	0.873	0.716	0.880	0.838	0.675
SSIM [29]	0.760	0.764	0.583	0.698	0.700	0.518
SSIM-CNN [51]	0.861	0.849	0.687	0.814	0.800	0.626
SUMMER [80]	0.808	0.809	0.647	0.745	0.746	0.582
VIF [39]	0.863	0.828	0.671	0.825	0.765	0.605
VSI [68]	0.919	0.921	0.744	**0.909**	0.908	0.716
*LCSA1*	0.912	0.898	0.742	0.877	0.857	0.688
*LCSA2*	0.889	**0.930**	**0.772**	0.899	**0.917**	**0.751**
*LCSA3*	0.901	0.918	0.768	0.859	0.885	0.725
*LCSA4*	**0.927**	0.919	**0.772**	0.901	0.885	0.725

**Table 6:** Distortion types used in the applied benchmark IQA databases (LIVE [22], TID2013 [23], TID2008 [24], and CSIQ [25]).

Abbreviation	Description	LIVE [22]	TID2013 [23]	TID2008 [24]	CSIQ [25]
AGN	additive Gaussian noise	🗸	🗸	🗸	🗸
ANC	additive noise in color components		🗸	🗸	🗸
SCN	spatially correlated noise		🗸	🗸	
MN	masked noise		🗸	🗸	
HFN	high-frequency noise		🗸	🗸	
IN	impulse noise		🗸	🗸	
QN	quantization noise		🗸	🗸	
FF	simulated fast fading Rayleigh channel	🗸			
GB	Gaussian blur	🗸	🗸	🗸	
GCD	global contrast decrement				🗸
DEN	image denoising		🗸		
JPEG	JPEG compression noise	🗸	🗸	🗸	🗸
JP2K	JPEG2000 compression noise	🗸	🗸	🗸	🗸
JGTE	JPEG transmission errors		🗸	🗸	
J2TE	JPEG2000 transmission errors		🗸	🗸	
NEPN	non-eccentricity pattern noise		🗸	🗸	
BLOCK	local block-wise distortions of different intensity		🗸	🗸	
MS	mean shift		🗸	🗸	
CC	contrast change		🗸	🗸	
CCS	change of color saturation		🗸		
MGN	multiplicative Gaussian noise		🗸		
CN	comfort noise		🗸		
LCNI	lossy compression of noisy images		🗸		
ICQD	image color quantization with dither		🗸		
CA	chromatic aberration		🗸		
SSR	sparse sampling and reconstruction		🗸		

**Table 7:** Comparison on LIVE’s [22] distortion types. SROCC values are given. The highest values are typed in bold, while the second highest ones are underlined.

	FSIM	FSIMc	IFS	MS-SSIM	SFF	VIF	VSI	LCSA1	LCSA2	LCSA3	LCSA4
AGN	0.965	0.972	**0.988**	0.973	0.986	0.986	0.984	0.976	0.961	0.962	0.965
FF	0.950	0.952	0.940	0.947	0.953	0.965	0.943	0.984	0.978	**0.988**	0.980
GB	0.971	0.971	0.967	0.954	0.975	0.973	0.953	0.978	0.989	**0.997**	0.996
JPEG	0.983	0.984	0.978	0.982	0.979	**0.985**	0.976	0.974	0.973	0.964	0.965
JP2K	0.972	0.970	0.969	0.963	0.967	0.970	0.960	0.952	0.969	0.967	**0.978**
All	0.963	0.965	0.960	0.951	0.965	0.964	0.952	**0.974**	0.962	0.969	0.970

**Table 8:** Comparison on TID2013’s [23] distortion types. SROCC values are given. The highest values are typed in bold, while the second highest ones are underlined.

	FSIM	FSIMc	IFS	MS-SSIM	SFF	VIF	VSI	LCSA1	LCSA2	LCSA3	LCSA4
AGN	0.897	0.910	0.938	0.865	0.907	0.899	**0.946**	0.908	0.932	0.925	0.925
ANC	0.821	0.854	0.854	0.773	0.817	0.830	**0.871**	0.846	0.854	0.853	0.857
SCN	0.875	0.890	0.934	0.854	0.898	0.884	0.937	0.908	**0.940**	0.933	0.915
MN	0.794	0.809	0.796	0.807	0.819	**0.845**	0.770	0.792	0.769	0.811	0.801
HFN	0.898	0.904	0.914	0.860	0.898	0.897	**0.920**	0.904	0.914	0.909	0.903
IN	0.807	0.825	0.839	0.763	0.787	0.854	**0.874**	0.574	0.795	0.790	0.728
QN	0.872	**0.881**	0.834	0.871	0.861	0.785	0.875	0.854	0.886	0.844	0.863
GB	0.955	0.955	0.966	0.967	**0.968**	0.965	0.961	0.954	0.956	0.959	0.970
DEN	0.930	0.933	0.918	0.927	0.909	0.891	**0.948**	0.917	0.937	0.913	0.937
JPEG	0.932	0.934	0.929	0.927	0.927	0.919	**0.954**	0.921	0.930	0.929	0.932
JP2K	0.958	0.959	0.961	0.950	0.957	0.952	**0.971**	0.950	0.965	0.957	0.953
JGTE	0.846	0.861	0.893	0.848	0.883	0.841	**0.922**	0.854	0.891	0.863	0.859
J2TE	0.891	0.892	0.901	0.889	0.871	0.876	**0.923**	0.909	0.916	0.913	0.916
NEPN	0.792	0.794	0.784	0.797	0.767	0.772	0.806	**0.826**	0.815	0.815	0.822
BLOCK	0.549	**0.553**	0.100	0.480	0.179	0.531	0.171	0.452	0.353	0.328	0.185
MS	0.753	0.749	0.658	**0.791**	0.665	0.628	0.770	0.554	0.678	0.455	0.620
CC	0.469	0.468	0.447	0.463	0.469	**0.839**	0.475	0.535	0.448	0.631	0.423
CCS	0.275	**0.836**	0.826	0.410	0.827	0.310	0.810	0.712	0.829	0.813	0.813
MGN	0.847	0.857	0.879	0.779	0.843	0.847	**0.912**	0.875	0.900	0.882	0.875
CN	0.912	0.914	0.904	0.853	0.901	0.895	**0.924**	0.911	0.923	0.904	0.906
LCNI	0.947	0.949	0.943	0.907	0.926	0.920	0.956	0.951	**0.958**	0.945	0.957
ICQD	0.876	0.882	0.901	0.856	0.880	0.841	0.884	0.891	**0.903**	0.891	0.900
CA	0.872	**0.893**	0.886	0.878	0.879	0.885	0.891	0.862	0.873	0.870	0.874
SSR	0.957	0.958	0.956	0.948	0.952	0.935	0.963	0.948	0.957	**0.965**	0.955
All	0.802	0.851	0.870	0.785	0.851	0.677	0.894	0.788	**0.903**	0.821	0.823

**Table 9:** Comparison on TID2008’s [24] distortion types. SROCC values are given. The highest values are typed in bold, while the second highest ones are underlined.

	FSIM	FSIMc	IFS	MS-SSIM	SFF	VIF	VSI	LCSA1	LCSA2	LCSA3	LCSA4
AGN	0.857	0.876	0.917	0.809	0.873	0.880	**0.923**	0.887	0.916	0.906	0.905
ANC	0.853	0.893	0.896	0.805	0.863	0.876	**0.912**	0.887	0.890	0.893	0.889
SCN	0.848	0.871	0.931	0.821	0.894	0.870	0.930	0.894	0.915	**0.936**	0.918
MN	0.802	0.826	0.802	0.811	0.837	**0.868**	0.773	0.782	0.733	0.857	0.817
HFN	0.909	0.916	0.922	0.869	0.912	0.908	**0.925**	0.901	0.909	0.922	0.917
IN	0.745	0.772	0.814	0.691	0.748	**0.833**	0.830	0.396	0.729	0.752	0.618
QN	0.856	**0.873**	0.797	0.859	0.845	0.797	**0.873**	0.825	0.859	0.855	0.854
GB	0.947	0.947	0.960	0.956	0.962	0.954	0.953	0.933	0.944	0.953	**0.963**
DEN	0.960	0.962	0.949	0.958	0.938	0.916	**0.969**	0.936	0.956	0.964	0.963
JPEG	0.928	0.929	0.928	0.932	0.932	0.917	**0.962**	0.921	0.942	0.939	0.937
JP2K	0.977	0.978	0.978	0.970	0.977	0.971	0.985	0.975	**0.991**	0.986	0.977
JGTE	0.871	0.876	0.874	0.868	0.857	0.859	**0.916**	0.886	0.914	0.893	0.904
J2TE	0.854	0.856	0.878	0.861	0.839	0.850	0.894	0.889	0.885	**0.911**	0.901
NEPN	0.749	0.751	0.704	0.738	0.697	0.762	0.770	**0.831**	0.773	0.805	0.796
BLOCK	**0.849**	0.846	0.087	0.755	0.537	0.832	0.630	0.826	0.631	0.742	0.672
MS	**0.672**	0.655	0.522	0.734	0.523	0.510	0.671	0.460	0.383	0.554	0.497
CC	0.648	0.651	0.627	0.638	0.646	**0.819**	0.656	0.630	0.604	0.732	0.577
All	0.881	0.884	0.869	0.846	0.851	0.749	0.896	0.874	0.906	**0.921**	0.909

**Table 10:** Comparison on CSIQ’s [25] distortion types. SROCC values are given. The highest values are typed in bold, while the second highest ones are underlined.

	FSIM	FSIMc	IFS	MS-SSIM	SFF	VIF	VSI	LCSA1	LCSA2	LCSA3	LCSA4
AGN	0.926	0.936	0.959	0.947	0.947	0.958	0.964	0.965	0.971	0.967	**0.976**
ANC	0.923	0.937	0.953	0.933	0.955	0.951	0.964	0.912	0.948	0.962	**0.969**
GB	0.973	0.973	0.962	0.971	0.975	0.975	0.968	**0.983**	0.972	0.971	0.981
GCD	0.942	0.944	0.949	0.953	0.954	0.935	0.950	**0.975**	0.959	0.972	0.963
JPEG	0.965	0.966	0.966	0.963	0.964	0.971	0.962	0.967	**0.983**	0.981	0.979
JP2K	0.968	0.970	0.971	0.968	**0.976**	0.967	0.969	0.956	0.950	0.941	0.950
All	0.924	0.931	0.958	0.913	0.960	0.920	0.942	0.956	0.949	0.961	**0.973**

## Data Availability

In this paper, the following publicly available benchmark databases were used: 1. LIVE: https://live.ece.utexas.edu/research/quality/subjective.htm (accessed on 12 May 2022), 2. TID2013: http://www.ponomarenko.info/tid2013.htm (accessed on 12 May 2022), 3. TID2008: http://www.ponomarenko.info/tid2008.htm (accessed on 12 May 2022), and 4. CSIQ: https://isp.uv.es/data_quality.html (accessed on 12 May 2022).

## Abbreviations

The following abbreviations are used in this manuscript:

FR-IQA	full-reference image quality assessment
IQA	image quality assessment
KROCC	Kendall’s rank order correlation coefficient
MOS	mean opinion score
MSE	mean squared error
NR-IQA	no-reference image quality assessment
PLCC	Pearson’s linear correlation coefficient
PSNR	peak signal-to-noise ratio
RMSE	root mean square error
RR-IQA	reduced-reference image quality assessment
SA	simulated annealing
SROCC	Spearman’s rank order correlation coefficient
SSIM	structural similarity index
